# A Study From Saudi Arabia: What Do Patients Value When Choosing an Ophthalmic Surgeon?

**DOI:** 10.7759/cureus.94014

**Published:** 2025-10-07

**Authors:** Ibtisam Algouf, Khawlah Aldehailan, Renad AlSubaie, Mryam Al Najjar, Ghaida AlQarni, Bayan Abdullatif Alomair, Saif K Aldossari

**Affiliations:** 1 Internal Medicine, College of Medicine, King Faisal University, Al Ahsa, SAU; 2 College of Medicine, King Faisal University, Al Ahsa, SAU; 3 Medicine and Surgery, King Faisal University, Al Ahsa, SAU; 4 Family and Community Medicine, King Faisal University, Al Ahsa, SAU; 5 Medicine, King Faisal University, Al Ahsa, SAU; 6 Ophthalmology, King Faisal University, Al Ahsa, SAU

**Keywords:** eye surgery, ophthalmic surgeon, ophthalmologist, ophthalmology, saudi arabia, social media

## Abstract

Introduction: Patient involvement in healthcare decision-making is a growing trend, influenced by a variety of factors, including a surgeon’s reputation, experience, and digital presence. While much of the research has focused on general practitioners, there is limited literature on factors influencing patients’ choices of ophthalmic surgeons, particularly in Saudi Arabia.

Objective: This study aims to identify and assess the key factors influencing the selection of ophthalmic surgeons in Saudi Arabia.

Methodology: A cross-sectional, descriptive study was conducted using a validated online questionnaire. The sample consisted of 627 participants from Saudi Arabia, with a range of demographic characteristics. Descriptive and inferential statistical analyses were performed, including logistic regression to identify predictors of surgeon selection.

Results: The study found that the most influential factors in surgeon selection were the surgeon’s reputation (96.5%; 95% CI: 94.7-97.8%), qualifications (93.1%; 95% CI: 91.0-95.0%), and patient satisfaction (94.4%; 95% CI: 92.5-96.1%). Gender preference was less significant (30.1%; 95% CI: 27.0-33.4%), while social media presence had a limited impact (23.8%; 95% CI: 20.9-26.9%). Personal recommendations from friends and family (45.4%) and physicians (33.5%) played a significant role. Proximity to the hospital and cost also influenced decisions, with geographic accessibility being a key predictor.

Conclusion: The selection of ophthalmic surgeons in Saudi Arabia is primarily influenced by reputation, qualifications, and recommendations from trusted sources. Although social media and gender preferences were less influential, the findings highlight the complexity of patient decision-making and emphasize the importance of personalized care in a digital era. To facilitate more informed decision-making, healthcare institutions and professional associations could establish standardized platforms or databases that offer transparent access to surgeons' certifications and professional backgrounds.

## Introduction

Ophthalmic procedures are among the most commonly performed surgeries in developed countries. In the United States alone, approximately 3.9 million ophthalmic surgeries were carried out over a three-year period, ranging from routine cataract operations to more complex retinal procedures [1]. These figures reflect the growing demand for skilled ophthalmic surgeons and high-quality surgical care.

Enhancing the quality of healthcare services requires effective communication and trust between patients and physicians [2]. Over time, there has been a noticeable shift toward patient-centered care, where patients are more actively involved in healthcare decisions, including the selection of their treating surgeon. This involvement may influence not only their emotional well-being but also their overall surgical outcomes [3].

A previous systematic review highlighted the complex and varied considerations patients take into account when selecting a surgeon or hospital for surgical treatment [4]. These factors include clinical aspects such as the surgeon’s experience and reputation, as well as non-clinical elements like insurance coverage and convenience of access [5].

In recent years, many ophthalmologists have adopted social media platforms for professional purposes. These platforms are used for patient education, self-promotion, and enhancing patient engagement, factors that may also shape a patient’s choice of surgeon [6].

While several studies have examined the factors influencing patients' selection of surgeons for elective procedures such as orthopedic and bariatric surgeries [3,7,8], there is limited research focusing specifically on ophthalmology. To our knowledge, no studies have explored the determinants affecting patients’ choices of ophthalmic surgeons either globally or within Saudi Arabia. The unique cultural and religious context in Saudi Arabia may influence patient preferences for surgeon gender and nationality, highlighting the need to study local determinants.

Saudi Arabia has experienced significant development in healthcare infrastructure and ophthalmic services. A recent study found that one in seven medical students in the country is considering ophthalmology as a future career path [9]. With the increasing number of physicians entering the field, it is important to understand the factors that guide patients in selecting their ophthalmic surgeon.

Therefore, the aim of this study is to identify and assess the key factors influencing patients' choice of ophthalmic surgeons in Saudi Arabia. The study addresses the following research questions: (1) What factors most influence patients in Saudi Arabia when selecting an ophthalmic surgeon? (2) How do surgeon gender, nationality, and social media presence affect patient preferences?

## Materials and methods

Study design and setting

This study utilized a descriptive, cross-sectional observational design to explore factors influencing the selection of eye surgeons among adults in Saudi Arabia. Data were collected using a validated, self-administered online questionnaire adapted from a previously published instrument, with additional ophthalmology-specific items added to address factors relevant to surgeon selection [10].

Sample size and population

The required sample size was calculated using the Raosoft sample size calculator (Raosoft, Inc., Seattle, WA), based on a 95% confidence level, 5% margin of error, and an assumed 50% response distribution, resulting in a minimum sample of 385 participants [11]. The study targeted male and female residents of Saudi Arabia aged 18 years and above.

Data collection procedure

A structured questionnaire was developed using Google Forms (Google, Mountain View, CA) and distributed through popular online platforms, including WhatsApp and Twitter, to ensure a wide public reach. Participation was voluntary, and electronic informed consent was obtained prior to beginning the survey. No personally identifiable information was collected to maintain participant confidentiality. A convenience sampling approach was used, recruiting participants who accessed the questionnaire through online platforms.

Ethical considerations

Ethical approval was secured from the Research Ethics Committee at King Faisal University, Al Ahsa, Saudi Arabia (Reference No.: KFU-REC-2024-JUN-ETHICS1840). The study adhered to the ethical principles outlined in the Declaration of Helsinki.

Statistical analysis

A comprehensive statistical analysis was conducted on the dataset, encompassing both descriptive and inferential methodologies. Firstly, a descriptive analysis was conducted to summarize the participants' demographic characteristics, including age, gender, and other features. This provided an overview of the study population. Subsequently, inferential analyses such as binary logistic regression analysis were employed to determine the essential predictors that influence the decisions of patients undergoing surgery. ANOVA, independent t-test, and chi-square were used to check the score difference between variables. Statistical significance was established at a p-value of 0.05 or lower and a 95% confidence interval. All statistical analyses were executed using IBM's SPSS software, version 29.0.0 (IBM Corp., Armonk, NY).

## Results

The study aimed to investigate the factors influencing the selection of an eye surgeon among 627 participants (Table 1). A predominant proportion of participants were female, comprising 86.6% (n = 543), while males constituted 13.4% (n = 84). In terms of age, nearly half of the participants (46.9%, n = 294) were between 18 and 30 years old. Geographically, the majority of participants (65.1%, n = 408) resided in the Eastern region of Saudi Arabia. Regarding employment status, 39.7% (n = 249) were employed, while 34.0% (n = 213) were students. The most common income range among participants was 10,001 to 20,000 Saudi riyals (SAR), with 38.1% (n = 239) falling into this category.

**Table 1:** Sociodemographic characteristics of the participants. Table showing counts (n) and percentages (%) of gender, age, employment status, and monthly income.

Variable	Frequency (n = 627)	Percent
Gender		
Female	543	86.6
Male	84	13.4
Age		
18-30 years	294	46.9
31-40 years	104	16.6
41-50 years	145	23.1
51-60 years	62	9.9
>60 years	22	3.5
Employment status		
Unemployed/retired	165	26.3
Employee	249	39.7
Students	213	34.0
Monthly income (Saudi Riyal, SAR)		
<5000 SAR	45	7.2
5000-10000 SAR	158	25.2
10,001-20,000 SAR	239	38.1
>20,000 SAR	185	29.5

In terms of preferences and experiences regarding eye surgeons (Table 2), several key findings emerged. When asked about their preference for the gender of an eye surgeon, half of the participants (50.2%) expressed no preference. However, 34.1% preferred a male surgeon, and 15.6% preferred a female surgeon. Regarding nationality, a significant majority (60.8%) of participants preferred a Saudi surgeon, while 37.0% had no preference, and only 2.2% preferred a non-Saudi surgeon.

**Table 2:** Preferences and experiences of participants regarding eye surgeons. Table showing counts (n) and percentages (%) of preferred surgeon gender and nationality, and of prior eye procedures.

Variable	Frequency (n = 627)	Percent
Preferred gender of eye surgeon		
No preference	315	50.2
Female	98	15.6
Male	214	34.1
Preferred nationality of eye surgeon		
I don't care	232	37.0
I prefer non-Saudi	14	2.2
I prefer Saudi	381	60.8
Experience with eye-related surgery/procedure		
No	366	58.4
No (but planned)	88	14.0
Yes	173	27.6

Regarding past experiences with eye-related surgeries or procedures, 58.4% of participants reported having never undergone such procedures, 27.6% had previous procedures, and 14.0% had no prior surgeries but planned to undergo one.

**Figure 1:**
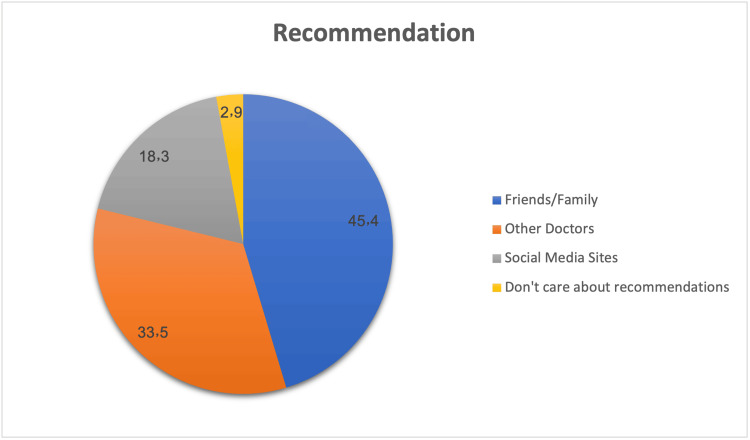
Sources of recommendations for choosing an eye surgeon. Pie chart showing percentages (%) of respondents citing each primary recommendation source (friends/family, other doctors, social media, and do not care about recommendations).

Figure 1 illustrates the sources of recommendations for choosing an eye surgeon. Friends and family were the most influential, with nearly half of the participants (45.4%) relying on them for advice. Doctors played a significant role, influencing 33.5% of participants, while social media had an unexpected but notable influence on 18.3% of participants. A small minority (2.9%) reported making their decision without any external recommendations.

**Figure 2:**
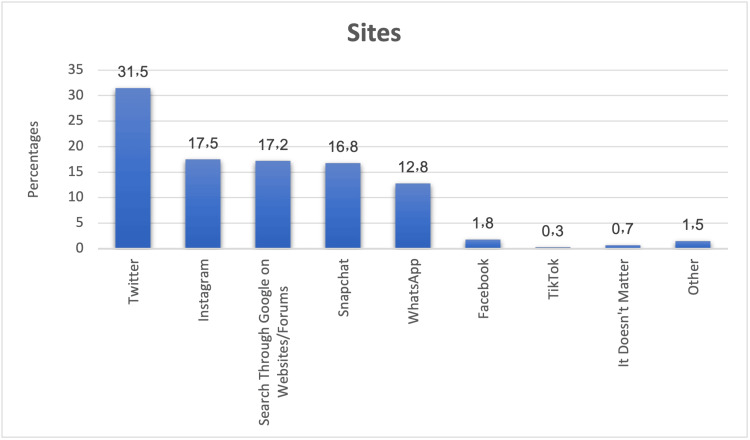
Influence of social media on the choice of an eye surgeon. Bar chart showing the distribution of platforms (Twitter, Instagram, Google search, Snapchat, WhatsApp, Facebook, and TikTok) reported to influence respondents’ surgeons as percentages (%).

Figure 2 depicts the influence of various social media platforms on the decision to choose an eye surgeon. Twitter emerged as the most influential platform, with 31.5% of participants considering it in their decision-making process. Instagram and Google searches followed, influencing 17.5% and 17.2% of participants, respectively. Snapchat had an impact on 16.8% of participants, while WhatsApp influenced 12.8%. Other platforms like Facebook, TikTok, and the perception that social media played no role had relatively minor influences.

Table 3 outlines the factors considered by participants when selecting an eye surgeon. The most highly valued factors were the surgeon’s reputation (96.5%), educational qualifications (93.1%), and patient satisfaction (94.4%). Practical aspects, such as appointment scheduling and waiting times (92.8%) and the financial cost of the operation (85.5%), were also deemed highly important. Surprisingly, the surgeon’s gender was less significant, with only 30.1% of participants considering it very important. Social media presence was regarded as unimportant by more than half of the participants (53.1%).

**Table 3:** Factors considered by participants when choosing an eye surgeon. Table showing counts (%) and percentages (%) for each Likert response across different factors (reputation, qualifications, gender, previous patient satisfaction, social media presence, appointment scheduling, cost, and insurance).

Factor	Not important	Neutral	Very important
Surgeon's reputation	3 (0.5%)	19 (3.0%)	605 (96.5%)
Surgeon's scientific qualifications	10 (1.6%)	33 (5.3%)	584 (93.1%)
Gender of the surgeon	283 (45.1%)	155 (24.7%)	189 (30.1%)
Meeting the needs and satisfaction of patients	9 (1.4%)	26 (4.1%)	592 (94.4%)
Social media appearance	333 (53.1%)	145 (23.1%)	149 (23.8%)
Arranging appointments and waiting lists	14 (2.2%)	31 (4.9%)	582 (92.8%)
Financial cost of the operation	30 (4.8%)	61 (9.7%)	536 (85.5%)
Insurance acceptance	75 (12.0%)	124 (19.8%)	428 (68.3%)

Table 4 shows the key predictors for selecting an eye surgeon among participants. Doctor's reputation significantly increased the likelihood of selection (B = 0.654, p = 0.011, Exp(B) = 1.923), while surgeon qualifications, social media presence, and other factors were non-significant. Proximity to the hospital was significant (B = -0.147, p = 0.038, Exp(B) = 0.864), but financial cost approached significance (B = -0.203, p = 0.065).

**Table 4:** Adjusted predictors for selecting an eye surgeon. Table showing results from a multivariable logistic regression model of factors associated with surgeon selection. Variables are presented with coefficient (B), p-value (Sig.), adjusted odds ratio (Exp(B)), and 95% confidence interval (95% CI). Significant predictors were doctor’s reputation (Exp(B) = 1.923, p = 0.011) and proximity to the hospital (Exp(B) = 0.864, p = 0.038).

Factor	B	Sig.	Exp (B)	95% CI (lower)	95% CI (upper)
Doctor’s reputation	0.654	0.011	1.923	1.160	3.189
Surgeon's scientific qualifications	-0.252	0.102	0.778	0.575	1.051
Gender of the surgeon	0.023	0.742	1.023	0.892	1.174
Meeting the needs of previous patients	-0.035	0.827	0.965	0.704	1.324
Social media appearance	0.080	0.320	1.083	0.925	1.268
Financial cost of the operation	-0.203	0.065	0.816	0.658	1.012
Arranging appointments and waiting lists	-0.056	0.698	0.945	0.712	1.256
Insurance acceptance	-0.022	0.788	0.979	0.836	1.145
Reputation of the hospital	0.087	0.547	1.091	0.821	1.450
Proximity to the hospital	-0.147	0.038	0.864	0.752	0.992

## Discussion

Globally, patient engagement has gained increasing attention due to its positive association with treatment adherence, follow-up compliance, and overall health outcomes, which contribute to reducing healthcare costs [12]. Technological advancements have facilitated this engagement, granting patients broader access to medical information through digital tools and online resources [13]. As a result, individuals are more empowered to participate in healthcare decisions, often researching procedures, treatment options, and provider credentials before committing to surgery [14].

Despite this progress, much of the existing literature has focused on patient preferences when selecting general practitioners rather than surgeons [15-17]. This gap is notable, as the criteria for choosing a surgeon may differ substantially due to the technical nature and risk profile of surgical care [3,7,8]. Among patients undergoing elective surgery, the selection of a treating surgeon is often seen as a critical decision, influenced by factors such as reputation, experience, and digital presence. Reputation, shaped by clinical outcomes, peer recognition, and qualifications, remains a key element, as does a surgeon’s experience with specific procedures. Recently, a surgeon’s social media activity, patient testimonials, and online visibility have become influential in shaping their perceived credibility and approachability [18].

In ophthalmology, despite the global volume of procedures performed annually, limited research has addressed the factors influencing patients’ decisions when choosing an ophthalmic surgeon. For example, between 2012 and 2014, an average of 1.3 million ambulatory ophthalmic surgeries were performed annually across 29 US states [1]. This study addresses this gap by examining how demographic characteristics, personal preferences, and both traditional and digital influences shape the selection of ophthalmic surgeons in Saudi Arabia.

Our findings revealed that gender plays a significant role in surgeon selection. While approximately half of the participants expressed no specific gender preference, males were more likely to prefer male surgeons, whereas females showed a greater inclination toward female surgeons for long-term care (p < 0.001). This aligns with studies by Abghari et al. and Imtiaz et al., which found that those with a preference often gravitated toward providers of the same gender [7,19]. Additionally, females in our study assigned more importance to practical elements like appointment scheduling and waiting times (p = 0.003), reflecting a tendency for females to prioritize convenience-based factors, while males emphasized qualifications and experience [18].

Nationality preference also emerged as a significant factor, with 60.8% of participants favoring Saudi surgeons. This may reflect cultural familiarity, linguistic ease, and trust in the local healthcare system. Personal recommendations were the most common source of surgeon selection (45.4%), followed by physicians (33.5%) and social media (18.3%). This contrasts with findings from Alosaimi et al., where recommendations from former patients were prioritized over those from friends or family [3], while a 2023 study in Qassim found recommendations less significant [10].

Digital influence was also notable, with Twitter being the most impactful platform (31.5%), followed by Instagram (17.5%). Previous studies have reported Snapchat’s influence, particularly for cosmetic procedures [20,21]. Alosaimi et al. found that two-thirds of patients valued social media reviews, and AlBalawi et al. reported that 73.1% of participants considered a surgeon’s social media presence essential [3,22]. In contrast, Coulter et al. suggested that media and advertisements had limited influence on patient decisions [23]. Age significantly affected social media importance, with younger participants (18-29 years) assigning less weight to it than older groups (p < 0.001).

When rating the importance of factors, 96.5% of participants deemed a surgeon’s reputation very important, followed by educational competencies, patient satisfaction, and healthcare facility reputation. These findings align with those of Alosaimi et al. and Aydin et al., who emphasized the importance of physician attitude and experience [3-5]. Logistic regression analysis confirmed the significance of reputation (p = 0.011) and proximity to the hospital (p = 0.038) in surgeon selection. Practical considerations like ease of appointment scheduling (92.8%), financial cost (85.5%), and insurance coverage (68.3%) were also highly influential. This is consistent with the findings of Fujita et al., who demonstrated that geographic accessibility improves healthcare utilization [24]. For ophthalmologists, these results highlight the practical importance of actively enhancing their reputation through patient-centered care, clear communication, empathetic interactions, and maintaining strong professional qualifications, as these factors directly influence patient choice and trust.

Economic status was a strong determinant of patient preferences. Lower-income groups were more likely to prioritize cost, insurance coverage, and even social media presence. Participants aged 41-50 years expressed the strongest concern about surgery’s financial burden (p < 0.001), consistent with the findings of Arpey et al., who noted the impact of socioeconomic status on healthcare access and outcomes [25].

Several study limitations, such as the gender imbalance in the sample and potential biases, should be considered when interpreting the results. Specifically, the majority of respondents were female and from the Eastern region, which may limit generalizability to the wider Saudi population. Future research can explore additional variables and further elucidate the dynamics influencing patients' decisions.

## Conclusions

This study provides valuable insights into the multifaceted factors influencing patients' choice of ophthalmic surgeons in Saudi Arabia. Key determinants included doctor reputation, accessibility, personal recommendations, and cost, as well as evolving elements such as gender preference and social media presence. The diversity in gender, age, and socioeconomic background among participants highlights the complexity of healthcare decision-making. Understanding these dynamics is essential for improving patient-centered care and guiding ophthalmic surgeons in fostering stronger relationships with their patients in an increasingly digital healthcare environment.

In light of these findings, it is recommended that healthcare institutions and professional associations implement policies ensuring transparent access to surgeons' qualifications, training, and practice history. Standardized platforms or databases could empower patients to make more informed decisions, ultimately enhancing trust and satisfaction within the healthcare system. Moreover, future studies could use longitudinal or experimental designs to establish causality between various factors and patients' ultimate choice of surgeon. These approaches would help clarify how different influences, such as reputation, accessibility, or recommendations, impact decision-making over time.

## Appendices

**Table 5:** Questionnaire - English version.

We are a group of female medical students from King Faisal University aiming to conduct research to measure the factors influencing the choice of an ophthalmic surgeon. Your participation is voluntary and will take only 2–4 minutes. Completing this questionnaire indicates your consent. No personal information will be collected.										
Age	18-30	31-40	41-50	51–60	61 and above					
Gender	Male	Female								
Region of residence	Eastern Region	Central Region	Western Region	Northern Region	Southern Region					
Occupation	Student	Employed	Unemployed	Retired						
Monthly household income (SAR)	Less than 5,000	5,000–10,000	10,001–20,000	Above 20,000						
What gender of ophthalmic surgeon do you prefer to visit frequently and for a long time?	Female	Male	No preference							
Do you prefer the ophthalmic surgeon performing your surgery to be Saudi?	Prefer Saudi	Prefer not Saudi	Doesn't matter							
Have you ever undergone a medical procedure or eye surgery?	Yes	No	No, but I plan to							
If yes, what was the procedure? (You may select more than one)	Vision correction laser	Cataract surgery	Glaucoma surgery	Eye tumor surgery	Keratoconus surgery	Cosmetic procedures (Botox or filler around the eyes)	Retinal laser	Intravitreal injection	Corneal transplant	
Are you a guardian of a child who needs or has needed eye surgery?	Yes	No								
Please rate the importance of the following factors when choosing an ophthalmic surgeon for you or your child:										
Choosing an ophthalmic surgeon: Recommendations source (You may select more than one)	Social media	Friends and family	Other doctors	Recommendations do not matter						
Choosing an ophthalmic surgeon: Doctor’s reputation	Very important	Somewhat important	Neutral	Slightly unimportant	Not important at all					
Choosing an ophthalmic surgeon: Surgeon’s scientific qualifications	Very important	Somewhat important	Neutral	Slightly unimportant	Not important at all					
Choosing an ophthalmic surgeon: Gender of the surgeon	Very important	Somewhat important	Neutral	Slightly unimportant	Not important at all					
Choosing an ophthalmic surgeon: Meeting the needs and satisfaction of patients	Very important	Somewhat important	Neutral	Slightly unimportant	Not important at all					
Choosing an ophthalmic surgeon: Social media appearance	Very important	Somewhat important	Neutral	Slightly unimportant	Not important at all					
Social media platforms that influence your choice. (You may select more than one)	Twitter	Snapchat	WhatsApp	Facebook	Instagram	Search through Google	Other			
Arranging appointments and waiting lists	Very important	Somewhat important	Neutral	Slightly unimportant	Not important at all					
Financial cost of the operation	Very important	Somewhat important	Neutral	Slightly unimportant	Not important at all					
Insurance acceptance	Very important	Somewhat important	Neutral	Slightly unimportant	Not important at all					
Reputation of the hospital	Very important	Somewhat important	Neutral	Slightly unimportant	Not important at all					
Proximity to the hospital	Very important	Somewhat important	Neutral	Slightly unimportant	Not important at all					

**Table 6:** Questionnaire - Arabic version.

نحن مجموعة طالبات من كلية الطب بجامعة الملك فيصل نهدف لعمل بحث لقياس العوامل المؤثرة في تحديد إختيار جراح العيون. مشاركتك في البحث طوعية وليست إجبارية وقد تستغرق ٢-٤ دقائق فقط واستكمالك للإستبيان دليل على موافقتك على المشاركة ولن يتم جمع أي معلومات عن هويتك الشخصية										
					٦١ وما فوق	٥١ - ٦٠	٤١-٥٠	٣١-٤٠	١٨-٣٠	العمر
								انثى	ذكر	الجنس
					المنطقة الجنوبية	المنطقة الشمالية	المنطقة الغربية	المنطقة الوسطى	المنطقة الشرقية	منطقة الاقامة
						متقاعد	غير موظف	موظف	طالب	الوظيفة
						اعلى من ٢٠،٠٠٠	١٠،٠٠١-٢٠،٠٠٠	٥٠٠٠-١٠،٠٠٠	اقل من ٥٠٠٠	دخل الاسرة الشهري
							لا يوجد تفضيل	ذكر	انثى	ما هو جنس جراح العيون الذي تفضل زيارته كثيرا ولفترة طويلة؟
							لا يهمني	أفضل ان لا يكون سعودي	أفضل ان يكون سعودي	هل تفضل ان يكون جراح العيون الذي سيعمل لك العملية سعودي؟
							لا ولكن اخطط لذلك	لا	نعم	هل سبق لك عمل اجراء طبي او عملية جراحية في العينين؟
	زراعة قرنية	حقن داخل العين	ليزر للشبكية	عمليات تجميلية (بوتكس او فيلير حول العينين)	عملية للقرنية المخروطية	عملية ورم في العين	عملية ماء ازرق (قلوكوما)	عملية ماء ابيض	ليزر تصحيح نظر	إذا الجواب السابق نعم، ماهي العملية/الاجراء الطبي؟ (يمكنك اختيار أكثر من اجابه)
								لا	نعم	هل انت ولي امر لطفل يحتاج او احتاج سابقا لعمل جراحة للعينين؟
										حدد مدى أهمية العوامل التالية في اختيارك لجراح العيون الذي سيعمل لك أو لطفلك عملية جراحية::
						لا تهمني التوصيات	أطباء اخرون	الأصدقاء والعائلة	التواصل الاجتماعي	عند اختيارك لجراح العيون تهمني التوصيات من خلال: (يمكنك اختيار أكثر من إجابة)
					لا يهم اطلاقا	لا يهم نوعا ما	حيادي	مهم نوعا ما	مهم جدا	عند اختيارك لجراح العيون: سمعة الطبيب:
					لا يهم اطلاقا	لا يهم نوعا ما	حيادي	مهم نوعا ما	مهم جدا	عند اختيارك لجراح العيون: الكفاءات التعليمية للجراح:
					لا يهم اطلاقا	لا يهم نوعا ما	حيادي	مهم نوعا ما	مهم جدا	عند اختيارك لجراح العيون: جنس الجراح:
					لا يهم اطلاقا	لا يهم نوعا ما	حيادي	مهم نوعا ما	مهم جدا	عند اختيارك لجراح العيون: تلبية الاحتياجات ومدى رضا المرضى السابقين
					لا يهم اطلاقا	لا يهم نوعا ما	حيادي	مهم نوعا ما	مهم جدا	عند اختيارك لجراح العيون: ظهوره على مواقع التواصل الاجتماعي
			غير ذلك	البحث عن طريق قوقل في المواقع والمنتديات	انستقرام	فيسبوك	واتس اب	سناب تشات	تويتر	مواقع التواصل الاجتماعي التي تؤثر على اختيارك لجراح العيون: (يمكنك اختيار اكثر من إجابة)
					لا يهم اطلاقا	لا يهم نوعا ما	حيادي	مهم نوعا ما	مهم جدا	عند اختيارك لجراح العيون: ترتيب المواعيد وأوقات الانتظار:
					لا يهم اطلاقا	لا يهم نوعا ما	حيادي	مهم نوعا ما	مهم جدا	عند اختيارك لجراح العيون: تكلفة العملية المادية
					لا يهم اطلاقا	لا يهم نوعا ما	حيادي	مهم نوعا ما	مهم جدا	عند اختيارك لجراح العيون: قبول التأمين
					لا يهم اطلاقا	لا يهم نوعا ما	حيادي	مهم نوعا ما	مهم جدا	عند اختيارك لجراح العيون: سمعة المستشفى أو المركز الصحي
					لا يهم اطلاقا	لا يهم نوعا ما	حيادي	مهم نوعا ما	مهم جدا	عند اختيارك لجراح العيون: قرب مسافة المستشفى أو المركز الصحي من مكان إقامتك
